# Pulmonary arterial mechanoreceptors mediate sustained sympathoexcitation during high altitude hypoxia in humans

**DOI:** 10.1113/EP093675

**Published:** 2026-02-15

**Authors:** Michiel T. Ewalts, Lydia L. Simpson, Jack S. Talbot, Elliott J. Jenkins, Travis D. Gibbons, Connor A. Howe, Lauren E. Maier, Emily R. Vanden Berg, Alex M. Williams, Philip N. Ainslie, Samuel J. Oliver, Craig D. Steinback, Mike Stembridge, Jonathan P. Moore

**Affiliations:** ^1^ Institute for Applied Human Physiology, Department of Sport and Exercise Sciences, College of Medicine and Health Bangor University Bangor UK; ^2^ Cardiff School of Sport and Health Sciences Cardiff Metropolitan University Cardiff UK; ^3^ Bristol Medical School University of Bristol Bristol UK; ^4^ Department of Sport Science, Performance Physiology and Prevention Universität Innsbruck Innsbruck Austria; ^5^ Cardiometabolic Health and Exercise Physiology Laboratory Baker Heart and Diabetes Institute Melbourne Australia; ^6^ Baker Department of Cardiometabolic Health, Melbourne Medical School University of Melbourne Melbourne Australia; ^7^ Centre for Heart, Lung, and Vascular Health University of British Columbia Okanagan Kelowna Canada; ^8^ Neurovascular Health Laboratory, Faculty of Kinesiology, Sport, and Recreation University of Alberta Canada; ^9^ Department of Cellular and Physiological Sciences, Faculty of Medicine University of British Columbia Vancouver Canada

**Keywords:** cardiovascular control, exercise, microneurography, MSNA, sympathetic activation

## Abstract

Sympathetic nervous system activation is a hallmark of high‐altitude hypoxia, yet the afferent mechanisms remain incompletely defined. We examined the relative contributions of pulmonary arterial mechanoreceptors and carotid chemoreceptors – two excitatory pathways co‐activated by hypoxia – to sustained sympathoexcitation at altitude. Nine healthy lowlanders (27 ±  7 years, three female) were studied after 6–9 days at 3800 m under four conditions: (1) control, (2) inhaled nitric oxide (iNO, 40 ppm) to reduce pulmonary arterial pressure, (3) low‐dose dopamine infusion (2 µg kg^−^^1^ min^−^^1^) to suppress the carotid chemoreflex, and (4) combined iNO and dopamine. End‐tidal oxygen and carbon dioxide were kept constant throughout. We assessed muscle sympathetic nerve activity (MSNA), systemic haemodynamics, ventilation and pulmonary arterial systolic pressure. iNO reduced pulmonary arterial pressure and significantly decreased MSNA (condition 1: 25 ± 8 bursts min^−1^ vs. condition 2: 21 ± 7 bursts min^−1^; *P* = 0.0415), whereas dopamine infusion reduced ventilation (*P* < 0.001) without a consistent effect on MSNA (condition 1: 25 ± 8 bursts min^−1^ vs. condition 3: 28 ± 13 bursts min^−1^; *P* = 0.112). Combined intervention produced a small reduction in sympathetic nerve activity (condition 3: 28 ± 13 bursts min^−1^ vs. condition 4: 26 ± 13 bursts min^−1^; *P* = 0.0643), likely due to baroreflex engagement. These findings confirm that unloading pulmonary arterial pressure attenuates MSNA, reinforcing the role of pulmonary mechanoreceptors in high altitude sympathoexcitation. Attempts to isolate a carotid chemoreflex contribution were likely confounded by dopamine's haemodynamic effect, which introduced variability and limited the specificity of this intervention. Thus, interpretation of this component remains exploratory, highlighting the integrative complexity of reflex control of high altitude sympathoexcitation in humans.

## Abstract (highlights)

- **What is the central question of this study?**
  Does high altitude sympathoexcitation arise from distinct contributions by pulmonary arterial mechanoreceptors and the carotid chemoreflex?
- **What is the main finding and its importance?**
  Reducing pulmonary arterial pressure with inhaled nitric oxide lowered sympathetic nerve activity and reset baroreflex control, confirming that pulmonary mechanoreceptors are key drivers of sympathetic activation at altitude. This insight could inform treatments for conditions with elevated pulmonary pressures. In contrast, attempt to suppress the carotid chemoreflex via systemic dopamine infusion did not alter sympathetic vasomotor outflow, highlighting the need for better methods to isolate its role.

## 1 INTRODUCTION

High altitude hypoxia is a powerful activator of the sympathetic nervous system (Fisher et al., 2018; Hansen & Sander, 2003; Lundby et al., 2018; Simpson et al., 2019; Tymko et al., 2023). While initially adaptive – supporting arterial blood pressure and oxygen delivery – sympathetic activity continues to rise even after arterial oxygen content normalises with acclimatisation (Schlittler et al., 2021; Simpson et al., 2024). Previously, we identified a mechanistic link between pulmonary arterial pressure, resulting from hypoxic pulmonary vasoconstriction, and increased muscle sympathetic nerve activity (MSNA) both at rest and during exercise (Simpson et al., 2020; Ewalts et al., 2026). This response is attributed to vagal afferent signalling from mechanosensitive receptors located at the pulmonary artery bifurcation (Bevan & Verity, 1962; Bianconi & Green, 1959; Coleridge & Kidd, 1960; Moore et al., 2004b). Pulmonary arterial mechanoreceptor activation may therefore contribute to sustained sympathoexcitation following restoration of arterial oxygen content (Hansen & Sander, 2003; Lundby et al., 2018; Mitchell et al., 2018; Xie et al., 2001). However, this signalling pathway has primarily been studied in humans alongside peripheral chemoreflex activation, another key hypoxia‐induced mechanism. Experimental separation of these pathways would offer novel insight into their relative contribution. Moreover, previous studies have not adequately controlled for changes in ventilation and arterial blood gases.

Peripheral chemoreceptors, specifically those located in glomus cells of the carotid and aortic bodies, are highly sensitive to changes in partial pressure of oxygen in the blood. Studies in experimental animals indicate a hyperbolic relationship between oxygenation and discharge frequency recorded from carotid chemoreceptors, with a threshold for greater responsiveness at around 70 mmHg (Vidruk et al., 2001), comparable to oxygen levels at an elevation of 2500 m. It is well established that peripheral chemoreceptor activation elicits dose–response increases in minute ventilation (Dempsey et al., 2014) and sympathetic outflow (Blumberg et al., 1980; Gregor & Jänig, 1977; Saito et al., 1988). Notably, the ventilatory and sympathoexcitatory responses to high altitude hypoxia have different time domains. A significant degree of ventilatory acclimatisation occurs within the first day of exposure to high altitude; however, the process is incomplete at that time, and acclimatisation continues for several days at least (Robbins, 2007). In contrast, persistent high altitude sympathoexcitation takes longer to develop, achieving a plateau at around 10 days, without any obvious further adaptation during exposure lasting up to 50 days (Lundby et al., 2018; Simpson et al., 2021).

The contribution of the peripheral chemoreflex to sustained sympathetic activation during high altitude acclimatisation remains uncertain. Two primary methods have been employed to suppress carotid chemoreceptor activity at altitude: hyperoxic breathing and low‐dose dopamine infusion. Hyperoxia has been shown to reduce MSNA modestly in acclimatised lowlanders, with effects largely mediated via reductions in heart rate rather than the probability of a burst (Fernandes et al., 2021; Hansen & Sander, 2003; Simpson et al., 2019; Roche et al., 2025). However, this approach is limited by potential confounding factors, including central chemoreceptor stimulation due to elevated arterial CO_2_, increased oxidative stress and direct neural effects of hyperoxia (Daristotle et al., 1991). Thus, the hyperoxia approach may underestimate the true contribution of the peripheral chemoreflex.

Alternatively, low‐dose intravenous dopamine has been used to attenuate carotid chemoreflex afferent signalling (Fisher et al., 2018). Dopamine acts on dopaminergic D1 and D2 receptors, with D2 receptors primarily mediating the effect on carotid sinus activity (Bisgard et al., 1980; Black et al., 1972; Ciarka et al., 2007). Notably, systemic dopamine cannot cross the blood–brain barrier (Bertler et al., 1966; Lorenzi et al., 1980) and thus does not affect the central chemoreceptors. This approach, therefore, is well‐established for modulating carotid chemoreflex activity in humans (Limberg et al., 2016; Niewinski et al., 2014). However, interpretation is complicated by dopamine's vasodilatory properties, which may elicit baroreflex‐mediated increases in MSNA (Fisher et al., 2018). Together, these limitations highlight the challenges in isolating the peripheral chemoreflex contribution to high altitude sympathoexcitation.

In this study, we sought to delineate the relative contributions pulmonary artery mechanoreceptor and carotid chemoreceptor signalling pathways during high altitude hypoxia. By conducting a series of targeted interventions, we aimed to uncouple pulmonary arterial mechanoreceptor feedback and the carotid chemoreflex in healthy lowland natives following approximately 1 week of terrestrial acclimatisation at 3800 m. To achieve this, we employed distinct experimental manipulations: inhaled nitric oxide (iNO) to unload pulmonary arterial mechanoreceptors, and low‐dose dopamine infusion to attenuate carotid chemoreceptor activity. We sought to minimise potentially confounding baroreflex‐mediated changes in MSNA using a dose of dopamine that previously had been reported not to elicit a vasodepressor effect at rest in healthy individuals (Stickland et al., 2011). In parallel, we utilised dynamic end‐tidal forcing to maintain relatively constant end‐tidal partial pressures of oxygen and carbon dioxide. Together, these interventions allowed us to isolate and evaluate the distinct neural contributions to elevated sympathetic activity at high altitude. In line with this, we hypothesised that unloading pulmonary arterial mechanoreceptors would reduce resting MSNA and reset the vascular arterial baroreflex, whereas suppression of the carotid chemoreflex would exert comparatively minimal effect.

## 2 METHODS

### 2.1 Ethical approval

The study was undertaken during the University of British Columbia (UBC) White Mountain Research Expedition 2022, which was approved by the Clinical Research Ethics Board of UBC (H22‐01091). All procedures conformed to the latest version of the *Declaration of Helsinki*, except for registration as a trial. All the procedures were explained verbally and in writing prior to obtaining signed informed consent.

### 2.2 Study design

The study utilised a counterbalanced design with two randomised infusion arms: low dose dopamine, to suppress the carotid chemoreflex, and volume matched saline control. Each infusion arm consisted of two breathing conditions: (1) dynamic end‐tidal forcing breathing ambient air, and (2) end‐tidal forcing with nitric oxide added to lower pulmonary arterial pressure and thus supress pulmonary arterial mechanoreceptor activity. Therefore, each participant performed four conditions with randomised infusion order: (1) saline infusion (Control), (2) NO (nitric oxide) inhalation during saline infusion (pulmonary mechanoreceptor unloading; iNO), (3) dopamine infusion (carotid chemoreflex suppression; Dopamine), and (4) NO inhalation during dopamine infusion (pulmonary arterial mechanoreceptor unloading combined with carotid chemoreflex suppression; Dopamine iNO). Based on previous findings (Simpson et al., 2020), an a priori power calculation indicated a minimum sample of 14 participants to detect an effect on MSNA burst frequency.

### 2.3 Study population

Fifteen volunteers (10 male, 5 female) were initially recruited as participants. All were members of the research expedition, lowland natives, non‐smokers, normotensive, with no self‐reported history of cardiovascular, respiratory, metabolic or neurological disease. Furthermore, all were free of respiratory infection, and none were taking over‐the‐counter prescription medicine on the day of study. All participants rapidly ascended to high altitude (White Mountain, CA, USA, 3800 m above sea level, barometric pressure 481 ± 3.2 mmHg) over the course of 9–10 h by motor vehicle. All participants were studied between 6 and 9 days following arrival at altitude. As is common with field research expeditions, study participants took part in other investigations, each addressing an a priori research question. As such, we ensured no overlap with the present study by allowing appropriate washout periods between studies.

On the day of testing, participants completed the Lake Louise acute mountain sickness (AMS) questionnaire (Roach et al., 2018) to evaluate symptoms of AMS. All participants reported a score ≤1 and therefore did not have clinically defined AMS. A suitable MSNA signal could not be obtained in three participants and the MSNA signal was lost before completing all testing procedures in three other participants. Thus, data from nine participants (3 females, 1 tested in the mid‐follicular phase, 1 in the late‐luteal phase cycle, and 1 in the low‐hormone phase of oral contraceptive use, all by self‐report) were included in the final analysis. Mean values for age, anthropometric parameters and resting blood pressure for this group of nine are presented in Table 1.

**TABLE 1:** Participant characteristics.

Characteristic	Value
Age (years)	27 ± 5
Height (cm)	176 ± 8
Body mass (kg)	71 ± 8
BMI	23 ± 3
Systolic blood pressure (mmHg)	118 ± 11
Diastolic blood pressure (mmHg)	82 ± 10
Mean arterial pressure (mmHg)	94 ± 9

### 2.4 Instrumentation, procedures, and data collection

**FIGURE 1:**
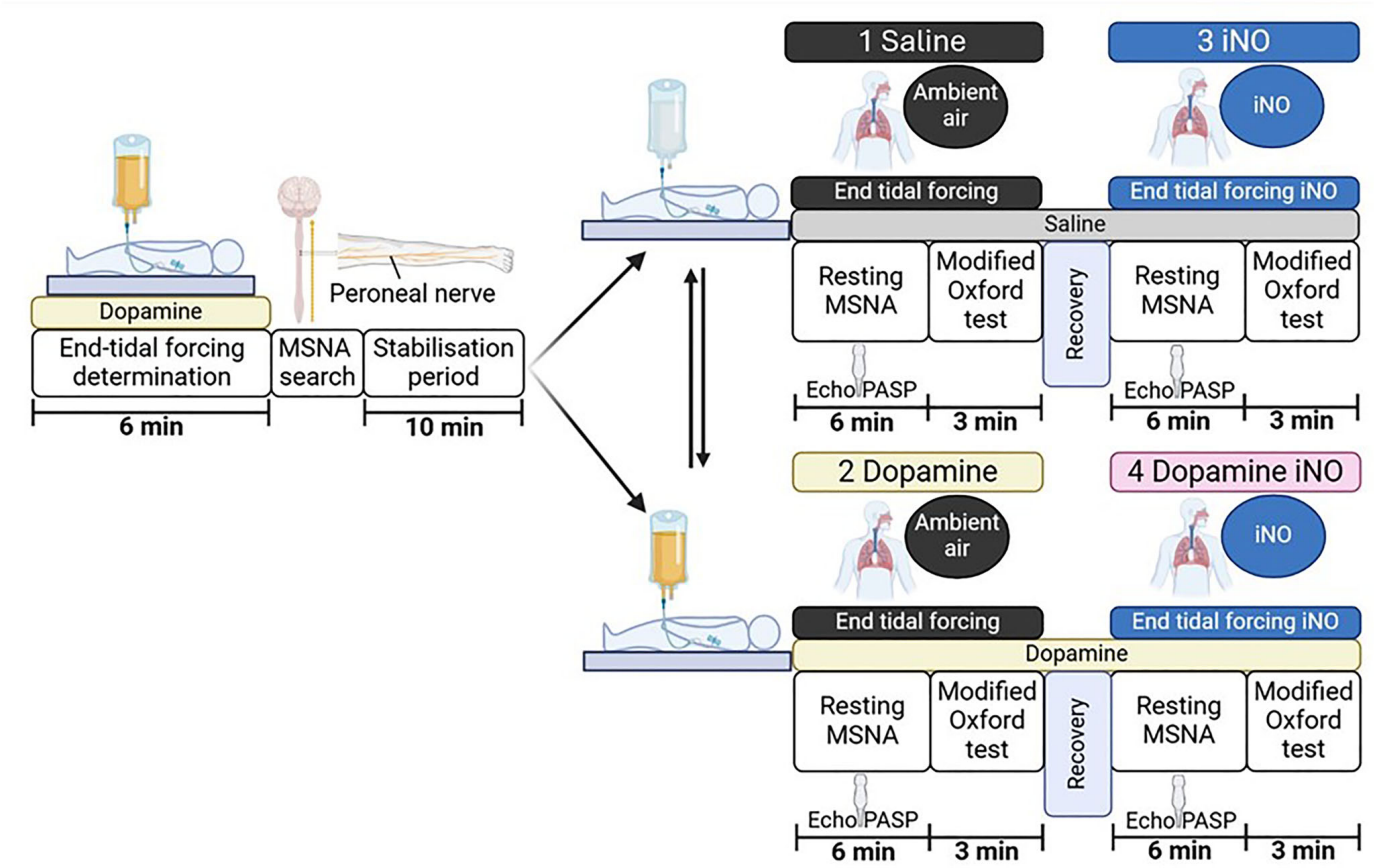
Experimental design. iNO, inhaled nitric oxide; MSNA, muscle sympathetic nerve activity; PASP, pulmonary arterial systolic pressure.

Figure 1 illustrates the sequence for an individual study. All testing was performed in a single session, commencing at least 4 h after a light meal. Participants were asked to abstain from caffeine, alcohol and vigorous exercise for at least 12 h before the appointed session. Following arrival at the laboratory, participants rested in the supine position. An intravenous catheter (20G Venflon, Becton, Dickinson and Company, Franklin Lakes, NJ, USA) was inserted into an antecubital vein for the purpose of administering infusions of dopamine and saline. Each participant was instrumented for continuous physiological monitoring and measurements: heart rate (three‐lead electrocardiogram; ML132, ADInstruments, Colorado Springs, CO, USA), arterial blood pressure (finger photoplethysmography; Finometer Pro, Finapres Medical Systems, Enschede, The Netherlands) and peripheral oxygen saturation ($S_{pO_{2}}$; fingertip pulse oximetry Choice Mmed, MD300C2, Beijing Choice Electronic Technology Co. Ltd, Beijing, China). A mouthpiece connected to a breath‐by‐breath respiratory gas analysis system consisting of a pneumotachograph (HR 800L; Hans Rudolph, Shawnee, KS, USA), a differential pressure amplifier (ML141, ADInstruments) and gas analyser (ML206; ADInstruments) enabled measurement of end‐tidal pressures of CO_2_ and O_2_, tidal volume (*V*_T_), breathing frequency (*f*_B_), and minute ventilation (${\dot{V}}_{E}$) using specifically designed software (LabVIEW, National Instruments, Austin, TX, USA).

Following cannulation and instrumentation, low‐dose dopamine (2 µg kg^−1^ min^−1^) was infused systemically (PHD ultra syringe pump, 33 DDS, Harvard Apparatus, Holliston, MA USA), for a minimum of 6 min, to suppress the effect of hypoxia on carotid chemoreceptors. Dopamine has a half‐life of around 2 min and after dopamine infusion was stopped, a wash‐out period of at least six half‐lives was used before continuation of the experimental protocol.

During infusion of dopamine, participants wore a nose clip and breathed room air through the mouthpiece for the purpose of establishing end tidal oxygen and carbon dioxide values for subsequent dynamic end tidal forcing. End‐tidal forcing was used to maintain consistent arterial blood gas partial pressures across all conditions of the protocol, despite ventilation changes induced by carotid chemoreflex suppression and potential alterations during nitric oxide inhalation (Simpson et al., 2020). Target end‐tidal values were derived during dopamine infusion, when oxygen was lowest and carbon dioxide highest.

Next followed a systematic search of the peroneal (common fibular) nerve for an acceptable MSNA signal, undertaken by an experienced microneurographer (J.M./C.S.). A tungsten microelectrode (200 µm diameter, 30 mm long, tapered to a 1–5 µm uninsulated tip) was inserted percutaneously into the nerve, with an uncoated tungsten reference electrode inserted subcutaneously 1–3 cm from the recording site. The recording electrode was manipulated until a characteristic pulse‐synchronous burst pattern was identified. The MSNA signal was confirmed by response to end‐expiratory apnoea but not to startle stimuli or skin stroking (Delius et al., 1972). The raw nerve signal was acquired (Neuroamp EX Headstage, ADInstruments), amplified (1000× pre‐amplifier and 100× variable gain isolated amplifier), band pass‐filtered (700–2000 Hz), rectified, and integrated (decay constant 0.1 s) to produce a mean voltage neurogram (LabChart Pro v8.3.1, ADInstruments). No adverse events or complications occurred during or following the microneurography procedure in any subject.

Following acquisition and stabilisation of an acceptable MSNA signal, saline or dopamine was infused in a randomised order. Five participants received saline first. Within each infusion arm of the protocol, the two breathing conditions were applied in sequence. After 1 min of infusion and breathing room air through the mouthpiece, participants were transferred to a dynamic end tidal forcing system (Air Force) which delivers gas to an inspiratory reservoir through a mixing and humidification chamber (Tymko et al., 2015). End tidal steady state was reached once values were within 1 mmHg of the desired target point for at least three consecutive breaths. Nitric oxide inhalation was always performed second during each infusion arm. By deferring nitric oxide inhalation, total infusion time was shortened because there was no requirement to wait for pulmonary arterial pressure to return to baseline between tests within the same infusion arm. Each breathing condition lasted for approximately 9 min and consisted of 6 min of steady‐state data acquisition followed by a modified Oxford test to assess arterial baroreceptor reflex responsiveness. A modified Oxford test consisted of bolus intravenous injection of sodium nitroprusside (SNP), followed 90 s later by phenylephrine (PE) to rapidly decrease and increase arterial pressure, respectively, as described previously (Simpson et al., 2019). A recovery period of approximately 8 min was allowed after the modified Oxford test of the first breathing condition. Participants continued to receive saline or dopamine infusion throughout the recovery, but they were able to breathe room air without the use of a mouthpiece or nose clip. Before nitric oxide inhalation, gas cylinders feeding the end‐tidal forcing system were switched from nitrogen to a speciality grade gas mixture comprising 50 parts per million (ppm) nitric oxide balanced in nitrogen (Linde plc, Danbury, CT, USA) titrated with 100% oxygen (Linde plc), to obtain a gas mixture containing 21% O_2_, 79% N_2_ and 40 ppm nitric oxide. As in previous work (Ewalts et al., 2026; Simpson et al., 2020), we utilised nitric oxide inhalation to study the effect of pulmonary arterial mechanoreceptors on MSNA and the vascular sympathetic baroreflex. This methodological approach harnesses the ability of nitric oxide to diffuse rapidly across the alveolar epithelial membrane into the pulmonary vascular smooth muscle, where it induces vascular smooth muscle relaxation (Steudel et al., 1999). Furthermore, any nitric oxide that diffuses into the intravascular space rapidly binds to haemoglobin, which serves to inactivate NO (Rimar & Gillis, 1993), thus avoiding systemic vasodilation (Frostell et al., 1991). Consequently, iNO acts to selectively unload pulmonary arterial mechanoreceptors and enables a confident interpretation of the origin of physiological effects discussed herein. The first infusion arm stopped at the end of each nitric oxide inhalation condition. The second infusion arm commenced after participants had recovered, which was determined by pulmonary arterial pressure returning to baseline. Table 2 summarises infusion durations, drug doses and pressure swings.

**TABLE 2:** Infusion duration and drug dosages and pressure changes during modified Oxford tests.

	Saline		Dopamine	
Total infusion duration (min)	32 ± 11		31 ± 11	
SNP dose (µg kg^−1^)	1.3 ± 0.2		1.3 ± 0.2	
PE dose (µg kg^−1^)	3.3 ± 0.3		3.3 ± 0.3	
	Control	iNO	Dopamine	Dopamine iNO
SNP DBP decrease (mmHg)	−9 ± 1	−8 ± 2	−8 ± 3	−11 ± 9
PE DBP increase (mmHg)	13 ± 5	12 ± 7	12 ± 7	12 ± 7
DBP change	21 ± 5	20 ± 9	20 ± 9	23 ± 15

DBP, diastolic blood pressure; iNO, inhaled nitric oxide; PE, phenylephrine; SNP, sodium nitroprusside.

Echocardiography was used to assess pulmonary artery systolic pressure (PASP), performed with a 1.5–4.5 MHz phased array transducer using a commercially available ultrasound system (GE Vivid Q, GE Healthcare, Milwaukee, WI, USA) and stored for subsequent off‐line analysis. Images were acquired from parasternal long‐ and short‐axis and apical four‐ and five‐chamber views in line with the American Society of Echocardiography guidelines for the assessment of the right and left heart (Lang et al., 2015, 2005; Rudski et al., 2010) by an experienced sonographer (M.S., A.W.). PASP was quantified as the maximum systolic pressure gradient across the tricuspid valve in line with the guidelines of the American Society of Echocardiography (Rudski et al., 2010). To derive pressure, the modified Bernoulli equation (4*v* ^2^) was applied to the peak systolic regurgitation jet velocity measured via continuous wave Doppler (Rudski et al., 2010). PASP measurement was undertaken at the midpoint of MSNA assessment periods under each condition. This timing was selected to ensure that both variables were captured under a stable physiological state, minimising the influence of transient haemodynamic fluctuations. Standardising the measurement point across participants further enhances comparability and internal validity, while maintaining methodological rigour consistent with protocols employed in our previous recent investigations utilising iNO administration (Ewalts et al., 2026; Simpson et al., 2020).

### 2.5 Data analyses

All data were sampled at 10 kHz using a commercial data acquisition software (LabChart Pro v8.3.1, ADInstruments) and stored for offline analysis. All continuous haemodynamics, MSNA measurements and respiratory measurements were averaged over the entire 6 min of each condition before a modified Oxford baroreceptor test. Finger photoplethysmography‐derived blood pressure waveforms were calibrated against the average of two brachial artery blood pressure measurements (Omron Healthcare, Milton Keynes, UK), taken before each condition. Systolic blood pressure (SBP), diastolic blood pressure (DBP) and mean arterial pressure (MAP), determined from maximum, minimum and mean values, were calculated on a beat‐by‐beat basis from the finger arterial pressure waveform. Multi‐unit bursts of integrated MSNA were identified using an automated detection algorithm (Chart Pro 8.3.1) and confirmed using established criteria (White et al., 2015) by a trained observer (M.E.), who performed data analysis whilst blinded to the condition. To account for differences in microelectrode position, burst amplitude data were normalised by assigning a value of 100 to the largest burst observed during baseline (White et al., 2015). All other bursts were calibrated against this value. Resting MSNA was quantified as burst frequency (burst min^−1^), mean burst amplitude (au.), total activity (mean burst amplitude × burst frequency [au min^−1^]), burst incidence (burst 100 HB^−1^), and total MSNA (mean burst amplitude × burst incidence [burst 100 HB^−1^]) (White et al., 2015). Amplitude indicates burst strength, whereas frequency and total activity reflect the amount of sympathetic vasoconstrictor outflow. Burst incidence and total MSNA are independent of time and reflect baroreflex ‘gating’ of bursts of sympathetic vasoconstrictor activity (Charkoudian & Wallin, 2014).

### 2.6 Baroreceptor reflex tests

Baroreflex control of MSNA (vascular sympathetic baroreflex) was estimated by calculating the slope (gain) of the linear relationship between DBP and (1) MSNA burst probability and (2) total MSNA during a modified Oxford test. In accordance with recently published guidelines (Holwerda et al., 2021), DBPs were aggregated into 3 mmHg bins and statistically weighted for the number of cardiac cycles in each bin to reduce the impact of non‐baroreflex variability (e.g., respiration). The percentage of cardiac cycles associated with a burst of MSNA (ranging from 0% to 100%) was calculated for each bin to calculate values for MSNA burst probability. To determine the relationship between diastolic pressure and total MSNA, the sum of normalised burst amplitudes for each bin was determined. This value was then divided by the number of bursts within that corresponding bin, to calculate mean burst amplitude (Usselman et al., 2015). Mean burst amplitude was then multiplied by the burst probability to calculate total MSNA. The slope of the linear relationship was determined by weighted linear regression analysis. Only slopes with (1) at least five data points and (2) *R* ≥ 0.5 were included in the data analysis. This resulted in eight comparisons of MSNA burst probability and five comparisons in total MSNA.

### 2.7 Statistical analyses

All data are presented as means ± standard deviation (SD). The statistical approach was designed to directly test the study's hypotheses through targeted comparisons: (1) effect of pulmonary arterial mechanoreceptor unloading on sympathetic nerve activity (comparing Control vs. iNO); (2) effect of suppression of the peripheral chemoreflex on sympathetic nerve activity (comparing Control vs. Dopamine); and (3) to isolate the vascular effect of dopamine, the effect of unloading of pulmonary arterial mechanoreceptors with suppressed peripheral chemoreflex was compared with Dopamine rather than Control (comparing Dopamine vs. Dopamine + iNO). Figures are arranged with Control positioned between iNO and Dopamine to make individual responses visually comparable across conditions. Differences between conditions were assessed using Student's paired *t*‐test (GraphPad Prism, Version 8.3.0, GraphPad Software, San Diego, CA, USA). A one‐tailed *t*‐test was applied when prior evidence supported a directional hypothesis for MSNA metrics (Fisher et al., 2018; Simpson et al., 2020), indicated on figures with an asterisk. For all other outcomes, repeated‐measures ANOVA was used to account for within‐subject variability and secondary factors, followed by Tukey's multiple comparisons test as required. Normality of distributions was confirmed using repeated Shapiro–Wilk tests. Statistical significance was set at *P* < 0.05, and effect sizes were reported as Cohen's *d* to quantify the magnitude of differences. *Post hoc* evaluation of end‐tidal gas measurements revealed inconsistencies affecting data from three participants. To maintain data integrity and avoid introducing bias, these data were excluded from all analyses. Subsequent checks confirmed that exclusion did not alter the primary outcomes or interpretation of the study.

## 3 RESULTS

### 3.1 Nitric oxide inhalation versus saline control

**FIGURE 2:**
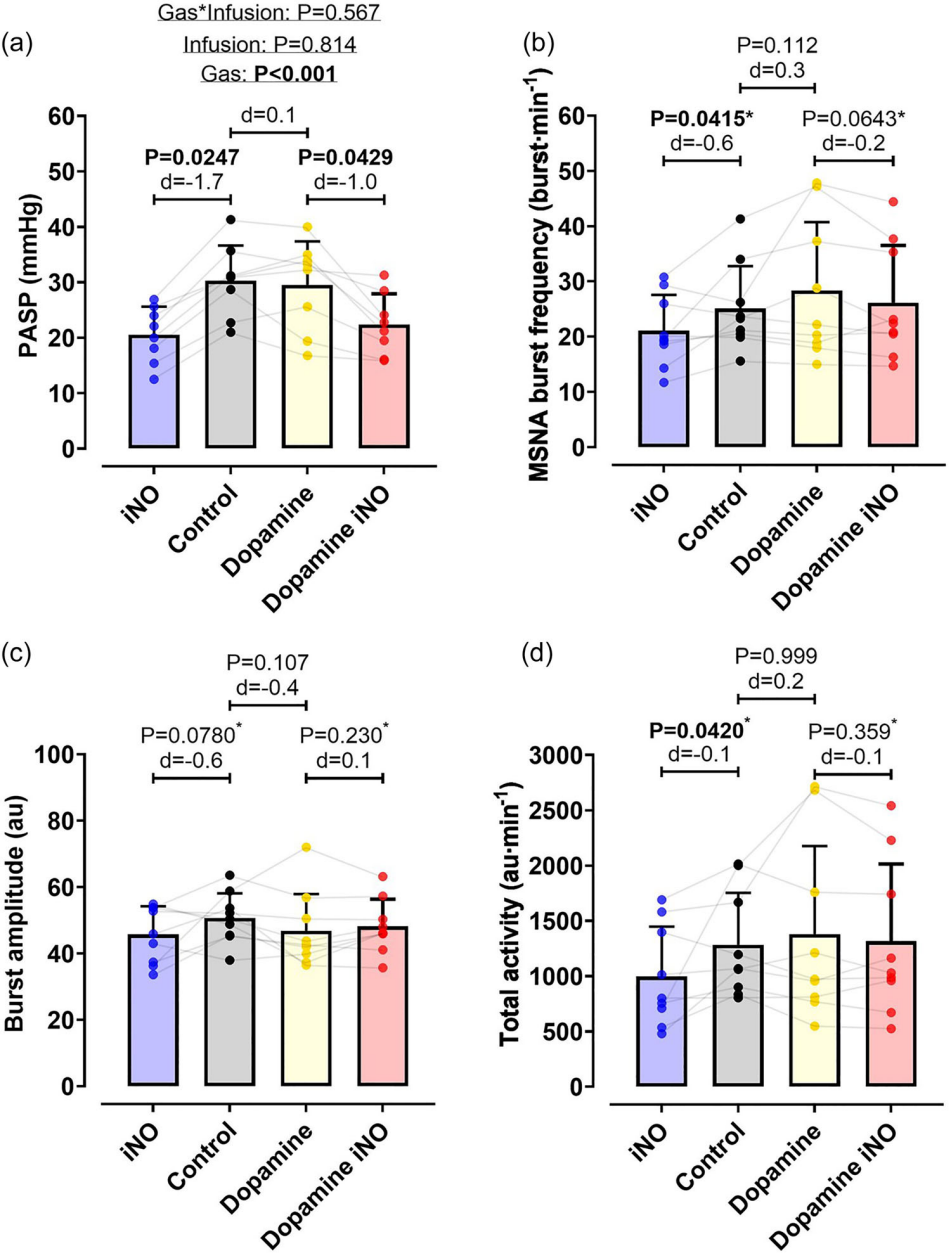
Group averages (*n* = 9) for pulmonary arterial systolic pressure (PASP, a), muscle sympathetic nerve activity (MSNA) burst frequency (b), mean burst amplitude (c), and total activity (d) under each condition. Comparisons used to test study hypotheses and corresponding condition order for visualisation are (1) Control vs. iNO – to assess the effect of unloading pulmonary arterial mechanoreceptors, (2) Control vs. Dopamine – to evaluate the effect of suppressing the peripheral chemoreflex, and (3) Dopamine vs. Dopamine + iNO – to isolate the vascular effects of dopamine while suppressing the peripheral chemoreflex and unloading pulmonary arterial mechanoreceptors. Statistical analysis in (a) consists of RM ANOVA and *post hoc* Tukey tests, while panels (b–d) consist of paired *t*‐tests, where the asterisk indicates a one‐tailed *t*‐test.

**FIGURE 3:**
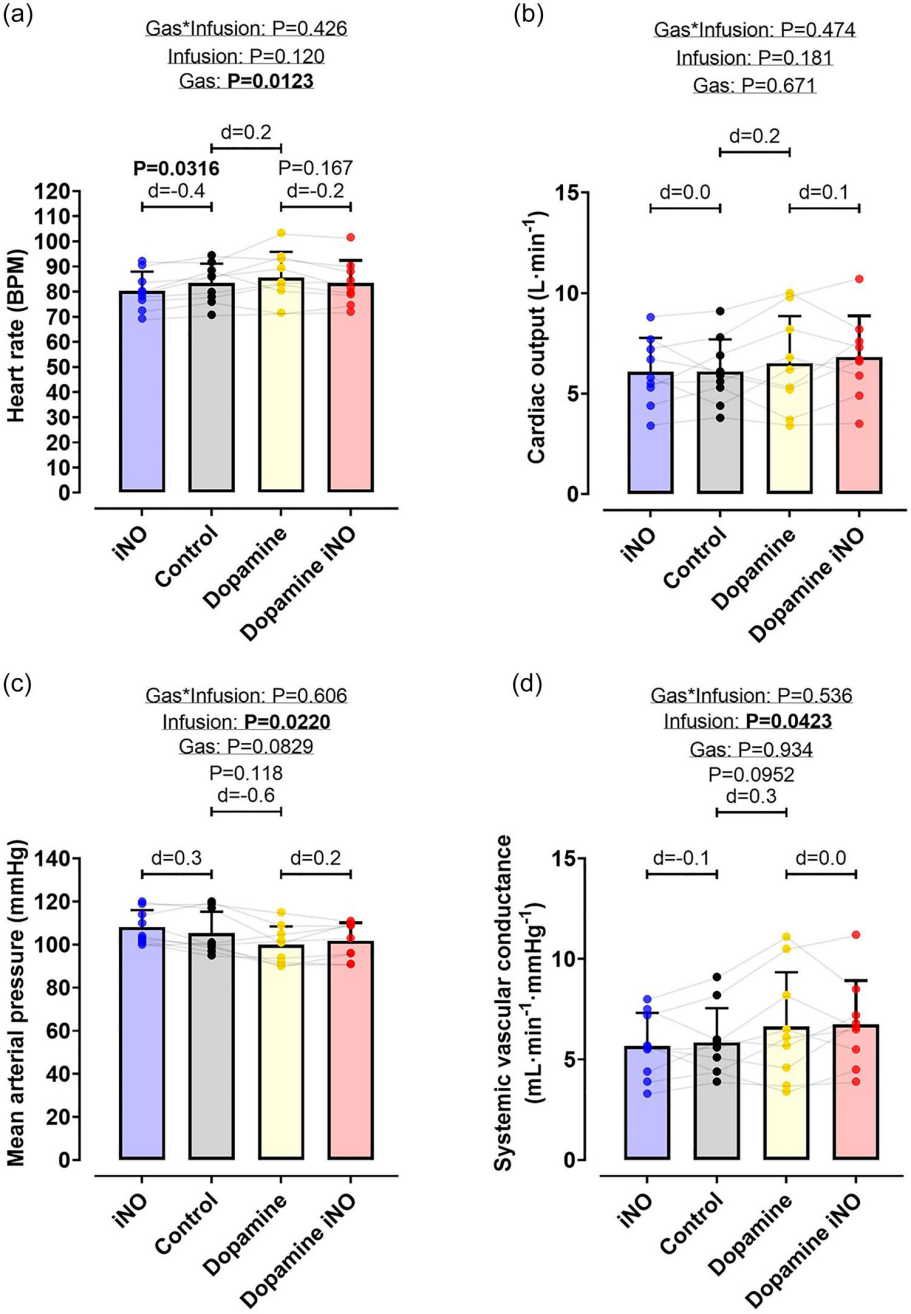
Group averages (*n* = 9) for heart rate (a), cardiac output (b), mean arterial pressure (c), and systemic vascular conductance (d). Comparisons used and corresponding condition order for visualisation are the same as for Figure 2. BPM, beats per minute. Statistical analyses consist of RM ANOVA and *post hoc* Tukey tests.

**FIGURE 4:**
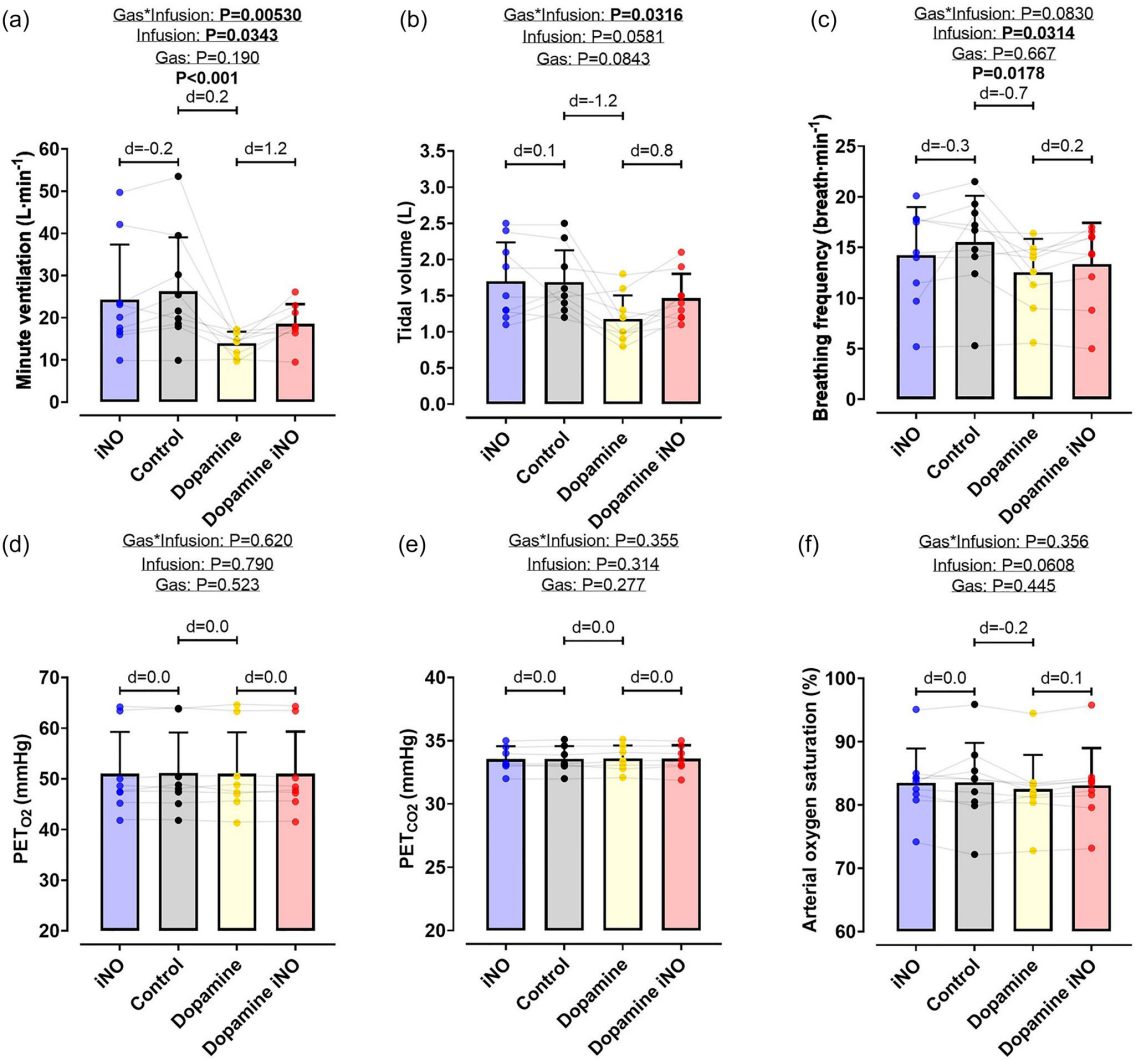
Group averages (*n* = 9) for minute ventilation (a), tidal volume (b), and breathing frequency (c), end tidal oxygen pressure (PET_O2_, *n* = 8) (d), end tidal CO_2_ pressure (PET_CO2_, *n* = 7) (e), and arterial oxygen saturation (f). Comparisons used and corresponding condition order for visualisation are the same as for Figures 2 and 3. Statistical analyses consist of RM ANOVA and *post hoc*Tukey tests.

**FIGURE 5:**
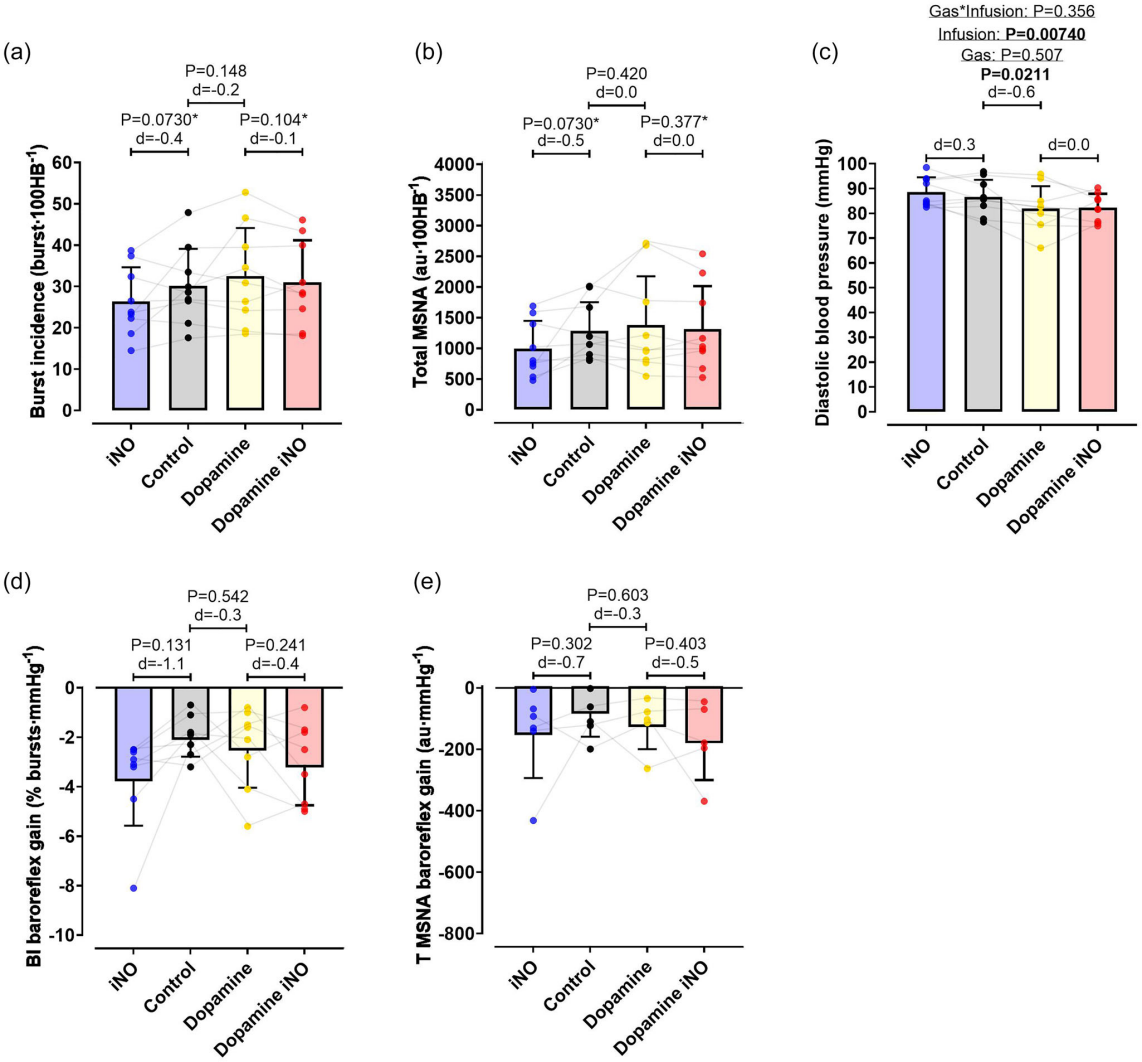
Group averages for indices of vascular sympathetic baroreflex function derived during a modified Oxford test. (a, b) Burst incidence (a) and total MSNA (b) are indices of baroreflex control of the occurrence (or probability) of a burst of activity. (c) Diastolic blood pressure. Baroreflex gain – slope of the linear relationship between DBP and MSNA burst probability (d, *n* = 8), and DBP and total MSNA (e, *n* = 5). Comparisons used and corresponding condition order for visualisation are the same as for Figures 2, 3, 4. Statistical analysis for (d) consists of RM ANOVA and *post hoc* Tukey tests, whereas analysis of data presented in all other panels consist of paired *t*‐tests, where * indicates one‐tailed *t*‐test.

Unloading of pulmonary arterial mechanoreceptors with iNO elicited a moderate reduction in MSNA burst frequency (*P* = 0.0415, *d *= −0.6) and a small reduction in total activity (*P* = 0.0420; *d* = −0.1; Figure 2). There was a moderate reduction in burst size amplitude that did not reach conventional significance (*P* = 0.0780, *d* = −0.6; Figure 2). Pulmonary mechanoreceptor unloading had no effect on ventilatory (Figure 4) and systemic haemodynamic parameters (Figure 3), apart from lower heart rate (*P* = 0.0316, *d* = −0.4). MSNA burst incidence and total MSNA – both indices of baroreflex control of the burst occurrence – were lower (small to moderate effect) without a change in DBP (Figure 5). This indicates that the vascular sympathetic baroreflex was reset to a lower burst occurrence when pulmonary arterial mechanoreceptors were unloaded. However, there was no effect on sympathetic baroreflex gain (slope), regardless of whether MSNA was quantified as burst probability (*n* = 8, Figure 5d) or total MSNA (*n* = 5; Figure 5e).

### 3.2 Dopamine infusion versus saline control

Suppression of carotid chemoreceptors by dopamine infusion had no effect on MSNA (burst frequency, burst amplitude and total activity). As expected, minute ventilation was lower during dopamine infusion compared with saline control (*P* < 0.001, *d* = −1.3; Figure 4a) mediated by a decrease in breathing frequency (*P* = 0.0178, *d* = −0.7, Figure 4c). Peripheral chemoreflex suppression had no effect on indices of vascular sympathetic baroreflex control (Figure 5), although DBP was lower (*P* = 0.0211, *d* = −0.6), indicating a leftward lateral shift in the vascular sympathetic baroreflex stimulus response relationship. This reduction suggests that dopamine exerted a vasodilatory effect, even at the low dose infused, resulting in baroreflex engagement.

### 3.3 Combined nitric oxide inhalation and dopamine infusion versus dopamine infusion

Unloading of pulmonary arterial mechanoreceptors combined with carotid chemoreflex suppression resulted in lower MSNA burst frequency, although this effect was small (*P* = 0.0643, *d *= −0.2 Fig, 2b). Burst amplitude (*P* = 0.230, *d* = 0.1, Figure 2c) and total activity (*P* = 0.359, *d* = −0.1 Figure 2d) were not different between conditions. Combined unloading of pulmonary arterial mechanoreceptors and peripheral chemoreflex suppression had no effect on diastolic pressure or any indices of vascular sympathetic baroreflex function (Figure 5).

## 4 DISCUSSION

The present study provides direct experimental support for a mechanistic link between pulmonary arterial pressure and sympathetic nervous system activation at high altitude. Specifically, three key findings emerged. First, reducing pulmonary arterial pressure – thereby reducing afferent input from pulmonary arterial mechanoreceptors – led to clear attenuation of high altitude sympathoexcitation, as reflected by reductions in MSNA burst frequency and amplitude. These findings support the concept that pulmonary mechanoreceptor afferents exert an excitatory influence on sympathetic outflow, and that their unloading contributes to resetting the vascular sympathetic baroreflex to a lower operating point. Second, pharmacological suppression of the peripheral chemoreflex via low‐dose dopamine infusion did not produce a uniform effect on sympathetic activity, despite a marked reduction in minute ventilation. This suggests that, under high altitude hypoxic conditions, chemoreflex modulation primarily influences respiratory drive rather than sympathetic regulation. The associated fall in DBP likely reflects dopamine's vasodilatory properties and consequent baroreflex engagement, which may have masked any direct sympathoinhibitory effect of chemoreflex suppression. Third, combined unloading of pulmonary arterial mechanoreceptors and chemoreceptor suppression yielded a smaller reduction in sympathoexcitation than pulmonary pressure lowering alone. This unexpected finding may reflect confounding engagement of the arterial baroreflex during dopamine administration, which could offset the sympathoinhibitory effects of pulmonary artery mechanoreceptor unloading. Thus, while the pulmonary mechanoreceptor contribution to sympathetic regulation at high altitude is strongly supported, conclusions regarding the chemoreflex component remain preliminary and warrant further investigation using alternative approaches that minimise baroreflex confounding.

### 4.1 Contribution of pulmonary arterial mechanoreceptors to high altitude sympathoexcitation

In response to lowering pulmonary arterial pressure, we demonstrated a significant reduction in basal sympathetic vasoconstrictor outflow, that is, quantity and strength of bursts. In addition, the probability of a burst of activity was lower without any change in diastolic pressure, which provides additional evidence to confirm that pulmonary arterial mechanoreceptors also modulate arterial baroreflex control of vasomotor output (Moore et al., 2011). Notably, the present study reiterates previous findings in humans at altitude (Simpson et al., 2020) and during hypoxic exercise (Ewalts et al., 2026). Further, dynamic end tidal forcing prevented a small rise in arterial oxygen saturation that was evident in our previous work at altitude. The influence of pulmonary arterial pressure was also evident when combined with carotid chemoreceptor suppression, although the effect was smaller, likely due to arterial baroreceptor reflex engagement during dopamine infusion. Together, these data indicate that an important neural signal for hypoxia‐induced sympathetic activation originates from mechanosensitive vagal nerve endings located in the pulmonary arterial system. This occurs in response to the increased pressure in the pulmonary arterial system due to hypoxic pulmonary vasoconstriction, which is a normal physiological response to a reduction in partial pressure of oxygen.

The effect of hypoxia on the pulmonary vasculature is time dependent. Pulmonary vascular tone increases rapidly within 5 min of hypoxic exposure (Talbot et al., 2005). This is followed by a plateau, lasting around 40 min, and slow intensification of the effect that extends to a maximal value over several hours (Dorrington et al., 1997). Furthermore, it appears that it takes a similar amount of time for pulmonary vascular tone to decrease following the restoration of normoxic breathing. Notably, prolonged altitude exposure leads to a sustained increase in pulmonary vascular resistance and pulmonary arterial pressure, both of which persist for up to 1 week after returning to normoxia (Groves et al., 1987; Hilty et al., 2016; Maufrais et al., 2017). Thus, this pattern of pulmonary haemodynamic response to prolonged sustained hypoxia could explain long‐lasting activation of sympathetic vasomotor outflow in humans that outlasts the stimulus to peripheral chemoreceptors (Hansen & Sander, 2003; Mitchell et al., 2018; Xie et al., 2001).

### 4.2 Contribution of peripheral chemoreceptors to high altitude sympathoexcitation

Herein, we have demonstrated that carotid chemoreflex suppression via dopamine infusion did not change basal sympathetic vasoconstrictor outflow. This is consistent with a previous report using a similar approach to blunting the responsiveness of the carotid chemoreceptors (Fisher et al., 2018). Our finding is also aligned with other studies showing that hyperoxia to silence peripheral chemoreceptors does not fully return MSNA to sea level/normoxic levels of activity (Hansen & Sander, 2003; Simpson et al., 2019). Although specific methods to assess the sensitivity and contribution of peripheral chemoreceptors have limitations (Simpson et al., 2024), the present data support the notion that peripheral chemoreceptors are not the primary origin of persistent high altitude sympathoexcitation (Fisher et al., 2018). However, we acknowledge that the depressor effect of dopamine limits this interpretation.

The sympathetic response to carotid chemoreceptor activation diverges from the ventilatory response within minutes of hypoxia exposure and this is maintained for several hours (Hunt et al., 2008; Keir et al., 2019; Prasad et al., 2020; Tamisier et al., 2007; van de Borne et al., 1998). This divergence indicates that sympathetic activity is influenced by factors other than carotid chemoreceptor activation during hypoxic breathing. In addition to the well‐established independent roles of the peripheral and central chemoreceptors, more current hypotheses on mechanisms of ventilatory acclimatisation incorporate newer and controversial concepts such as cardio‐ventilatory control based on plasticity of chemosensitivity, multiple sites of hypoxic sensing, interdependence of central and peripheral chemoreceptors, and upregulation of central nervous system neurons comprising respiratory and sympathetic regulatory pathways. Thus, regulation of sympathetic outflow and respiration is complex and integrative and may rely on multiple afferent inputs working in tandem. While the mechanistic influence of pulmonary arterial mechanoreceptors appears more prominent in sustaining sympathetic activation, the precise contribution of carotid chemoreceptor pathways remains to be fully elucidated. This contrasts with the well‐established and dominant contribution of peripheral chemoreceptors to ventilatory acclimatisation under hypoxic conditions (Getu et al., 2026; Powell, 2007). Further studies employing selective chemoreceptor inhibition (e.g., P2X3 receptor antagonist) are needed to clarify their role within the broader framework of autonomic adaptation to altitude.

### 4.3 Pulmonary arterial mechanoreceptor–respiratory activity coupling

The pulmonary circulation is a low‐pressure, high flow circuit that accommodates the entirety of right heart output (Naeije & Dedobbeleer, 2013). Notably, due to the anatomical position, pulmonary arterial mechanoreceptors are ideally situated to sense blood pressure variation within the pulmonary circulation arising from both upstream and downstream changes. This allows them to relay information to the central nervous system that could be used to adjust ventilation to match cardiac output and pulmonary perfusion (Wasserman et al., 1974). Studies in anaesthetised animals indicate that pulmonary arterial mechanoreceptor activation drives respiratory (phrenic nerve) activity (Kan et al., 1979; McMahon et al., 2000). Furthermore, pulsatile afferent activity arising from pulmonary arterial mechanoreceptors is most intense during the inspiratory phase of respiration (Moore et al., 2004a), presumably because transmural pressure is larger and the distending force is greater.

In the present study, we observed some contrasting effects of pulmonary mechanoreceptor unloading on respiratory activity. With peripheral chemoreflex activation intact, unloading pulmonary arterial mechanoreceptors (nitric oxide inhalation vs. control), produced no significant change in minute ventilation. When the carotid chemoreflex was suppressed, unloading pulmonary arterial mechanoreceptors coincided with a large estimated increase in minute ventilation (*d* = 1.2), although this effect did achieve statistical significance, reflecting limited statistical power rather than lack of physiological impact. These divergent effects when pulmonary arterial mechanoreceptors are unloaded is difficult to reconcile. Contrasting findings in animal models and conscious humans could be explained by differences in central respiratory drive (Moore et al., 2004a, 2004b, 2011). Respiratory depression is common during anaesthesia, and this may heighten the sensitivity of the respiratory centre to certain stimuli, including pulmonary mechanoreceptor afferent input. However, as demonstrated in the present study, low‐dose dopamine also depresses ventilation. Relative hyperventilation observed when pulmonary mechanoreceptors were unloaded by iNO in combination with carotid chemoreflex suppression was driven by greater tidal volume, rather than an increase in breathing frequency. This may reflect the bronchodilator action of nitric oxide, leading to increased airflow and improved ventilation, rather than a change in respiratory drive per se. In future studies, it will be important to properly explore the interaction of pulmonary arterial pressure and respiratory activity to establish if pulmonary arterial mechanoreceptor stimulation effects central respiratory drive.

### 4.4 Physiological relevance and clinical perspective

Pulmonary arterial mechanoreceptors have emerged as a significant neural input under conditions of chronic hypoxia, thus offering a mechanistic explanation for findings in several previous high‐altitude studies (Fisher et al., 2018; Hansen & Sander, 2003; Simpson et al., 2019). In a landmark microneurographic study conducted at a high altitude, Duplain and colleagues demonstrated a strong positive correlation between resting pulmonary arterial pressure and MSNA (Duplain et al., 1999). Importantly, individuals prone to high altitude pulmonary oedema (HAPE) exhibited markedly elevated sympathetic activation compared with HAPE‐resistant individuals, with firing rates approximately twice as high in the susceptible group. The present study, in conjunction with our previous work (Simpson et al., 2020), establishes a causal relationship between elevations in pulmonary arterial pressure and increased sympathetic nervous system activity under hypoxic conditions. This mechanistic insight may help explain the exaggerated MSNA observed in individuals predisposed to HAPE, as well as in patients with pulmonary or cardiac pathologies characterised by pulmonary hypertension and/or hypoxaemia (Plunkett et al., 2025; Velez‐Roa et al., 2004).

### 4.5 Limitations and other considerations

First, due to the nature of expedition research, our sample size was small. A priori power calculation estimated a required sample of 14. However, 9 of 15 recruited participants were studied fully. Subsequent power calculation indicated that the power for the pairwise comparison between Dopamine versus Dopamine + iNO – to isolate the vascular effects of dopamine while suppressing the peripheral chemoreflex and unloading pulmonary arterial mechanoreceptors – was 0.72. Thus, a sample of 11 participants was required to achieve statistical significance for this comparison. Second, the use of low‐dose dopamine to supress carotid chemoreflex input introduced interpretive challenges. In 7 of 9 participants, dopamine infusion reduced MAP. Thus, it is possible that engagement of the arterial baroreflex prevented clean isolation of carotid chemoreceptor control of sympathetic outflow. This confounding influence limited interpretation of MSNA changes as solely due to chemoreflex suppression. These findings raise important concerns about the suitability of dopamine as a tool for assessing chemoreflex contributions to sympathoexcitation, as even the low dose used in this study produced haemodynamic effects, contrary to previous reports (Stickland et al., 2011). Furthermore, there is no universally effective dose for chemoreflex inhibition in humans (Limberg et al., 2016) and preliminary titration is likely necessary to identify an optimal dose that suppresses chemoreflex activity without altering arterial pressure. Due to logistical constraints inherent to high altitude research, such titration was not feasible in the present study. Consequently, while our approach was conceptually innovative these physiological complexities hinder definitive mechanistic inference regarding the relative contribution of the carotid chemoreflex. Third, to minimise the length of time required for pulmonary arterial pressure to recover to baseline following nitric oxide inhalation, the order of end tidal forcing with and without nitric oxide inhalation was not randomised. Therefore, a potential order effect on the study findings cannot be excluded. Fourth, we observed that unloading pulmonary arterial mechanoreceptors coincided with a lower heart rate compared with control. This finding differs from a previous observation that pulmonary arterial mechanoreceptors do not influence heart rate (Simpson et al., 2020). The explanation for the difference is unclear. However, it may be related to experimental design, specifically time required to recover from the bolus of PE given during the modified Oxford test under saline control. Although approximately 8 min was allowed for clearance of the PE effect, heart rate at the start of nitric oxide inhalation was lower compared with saline control for 8 out of 9 participants. This suggests that the heart rate reduction observed when pulmonary arterial mechanoreceptors were unloaded by iNO was not a direct effect.

### 4.6 Conclusion

This present study provides robust confirmatory evidence that unloading pulmonary arterial mechanoreceptors attenuates MSNA and resets baroreflex control of vasomotor outflow, reinforcing the mechanistic role of pulmonary arterial mechanoreceptors in high altitude sympathoexcitation. This replication adds weight to previous findings and represents a key strength of the work. In contrast, the novel aim of isolating carotid chemoreflex contributions was constrained by methodological limitations, particularly the confounding haemodynamic effects of dopamine, which engaged the arterial baroreflex and introduced interpretive complexity. As such, conclusions regarding the relative contribution of the carotid chemoreflex should be considered preliminary. Future studies should employ alternative strategies – such as targeted pharmacological approaches – to achieve clean chemoreflex suppression without systemic haemodynamic interference. These refinements will enable more definitive mechanistic insights into the interplay between pulmonary arterial mechanoreceptors and carotid chemoreceptors in regulating sympathetic outflow at altitude.

## AUTHOR CONTRIBUTIONS

Conception or design of the work: Michiel T. Ewalts, Lydia L. Simpson, Mike Stembridge, Jonathan P. Moore. Preparation for the work, acquisition, analysis or interpretation of data: Michiel T. Ewalts, Samuel J. Oliver, Jack S. Talbot, Elliott J. Jenkins. Travis D. Gibbons, Connor A. Howe, Lauren E. Maier, Emily R. Vanden Berg, Philip N. Ainslie, Lydia L. Simpson, Craig D. Steinback, Mike Stembridge, Jonathan P. Moore. Drafting the manuscript: Michiel T. Ewalts, Jonathan P. Moore. Critically revising for important intellectual content: all authors. All listed authors have read and approved the final version of this manuscript and agree to be accountable for all aspects of the work in ensuring that questions related to the accuracy or integrity of any part of the work are appropriately investigated and resolved. All persons designated as authors qualify for authorship, and all those who qualify for authorship are listed.

## CONFLICT OF INTEREST

None declared.

## Supporting information

Statistical Summary Document

## Data Availability

The data that support the findings of this study are available from the corresponding author upon reasonable request.
