# Supplementary material for: Pulmonary arterial mechanoreceptors mediate sustained sympathoexcitation during high altitude hypoxia in humans
Source: Exp Physiol. 2026 Feb 15:10.1113/EP093675. Online ahead of print. doi: 10.1113/EP093675 (PMC13394917; doi:10.1113/EP093675)
Supplement: Supplementary file 1 — Statistical Summary Document [file EPH-9999-0-s001.docx]

**Manuscript Title: Pulmonary arterial mechanoreceptors mediate sustained sympathoexcitation during high altitude hypoxia in humans**

**Authors:** Michiel T Ewalts, Lydia L Simpson, Jack S Talbot, Elliott J Jenkins, Travis D Gibbons, Connor A Howe, Lauren E Maier, Emily R Vanden Berg, Alex M Williams, Philip N Ainslie, Samuel J. Oliver, Craig D Steinback, Mike Stembridge, Jonathan P Moore

**Animal model used, if applicable:** N/A

**Underlying hypothesis:** This investigation tests the hypothesis that unloading pulmonary arterial mechanoreceptors would reduce resting muscle sympathetic nerve activity (MSNA) and reset the vascular arterial baroreflex, whereas suppression of the carotid chemoreflex would exert comparatively minimal effect.

**Definitions of ‘n’:**

[Define ‘n’. If definitions differ, please indicate which definition applies to which experimental question number.]

n = number of subjects included in statistical analyses

**Statistical summary table:**

| Experimental question number* | Finding/ conclusion | Experimental location/ variable  e.g. cortex vs cerebellum or genotype | Mean value  (or other summary statistic) | SD | n (value) | P**  ANOVA | P**  Posthoc test | Units | Data comparisons  e.g. WT vs KO | Statistical test | Any other variable  e.g. subjects’ age or sex | Figure/table in which data are presented |
| --- | --- | --- | --- | --- | --- | --- | --- | --- | --- | --- | --- | --- |
| Question 1. Effect of iNO on PASP and cardiovascular haemodynamics | Inhalation of 40ppm nitric oxide significantly reduced PASP with no effect on peripheral oxygen saturation, heart rate, cardiac output, mean arterial pressure666 or systemic vascular conductance. | Pulmonary artery systolic pressure (PASP) | CON: 30 | 7 | 8 | - | - | mmHg | CON vs iNO | Two-way Anova  Post hoc: Tukey’s test | - | Figure 2 |
|  |  |  | iNO: 21 | 5 | 8 | P<0.001 | 0.0247 |  |  |  |  |  |
|  |  | Peripheral oxygen saturation (SpO_2_) | CON: 84 | 6 | 9 | - | - | % | CON vs iNO | Two-way Anova  Post hoc: Tukey’s test | - | Figure 4 |
|  |  |  | iNO: 84 | 5 | 9 | 0.445 | - |  |  |  |  |  |
|  |  | Heart rate (HR) | CON: 84 | 8 | 9 | - | - | Beats per minute (bpm) | CON vs iNO | Two-way Anova  Post hoc: Tukey’s test | - | Figure 3 |
|  |  |  | iNO: 80 | 8 | 9 | 0.0123 | 0.0316 |  |  |  |  |  |
|  |  | Cardiac output (Qc) | CON: 6.1 | 1.6 | 9 | - | - | Litres per min (L·min^-1^) | CON vs iNO | Two-way Anova  Post hoc: Tukey’s test | - | Figure 3 |
|  |  |  | iNO: 6.1 | 1.7 | 9 | 0.671 | - |  |  |  |  |  |
|  |  | Mean arterial pressure (MAP) | CON: 105 | 10 | 9 | - | - | mmHg | CON vs iNO | Two-way Anova  Post hoc: Tukey’s test |  | Figure 3 |
|  |  |  | iNO: 108 | 8 | 9 | 0.0829 | - |  |  |  |  |  |
|  |  | Systemic vascular conductance | CON: 5.9 | 1.8 | 9 | - | - | mL·min^-1^·mmHg | CON vs iNO | Two-way Anova  Post hoc: Tukey’s test | - | Figure 3 |
|  |  |  | iNO: 5.7 | 1.6 | 9 | 0.952 | - |  |  |  |  |  |
| Question 2. Effect of iNO on ventilation | Inhalation of 40ppm nitric oxide did not change ventilation or end tidal volumes | Ventilation (V_E_) | CON: 26 | 13 | 9 | - | - | Litres per min (L·min^-1^) | CON vs iNO | Two-way Anova  Post hoc: Tukey’s test | - | Figure 4 |
|  |  |  | iNO: 24 | 13 | 9 | 0.190 | - |  |  |  |  |  |
|  |  | Tidal volume (V_T_) | CON: 1.7 | 0.5 | 9 | - | - | Litres | CON vs iNO | Two-way Anova  Post hoc: Tukey’s test | - | Figure 4 |
|  |  |  | iNO: 1.7 | 0.5 | 9 | 0.0843 | - |  | CON vs iNO |  |  |  |
|  |  | Breathing frequency (F_B_) | CON: 16 | 5 | 9 | - | - | Breaths per minutes | CON vs iNO | Two-way Anova  Post hoc: Tukey’s test | - | Figure 4 |
|  |  |  | iNO: 14 | 5 | 9 | 0.667 | - |  |  |  |  |  |
|  |  | PET_O2_ | CON: 51 | 8 | 8 | - | - | mmHg | CON vs iNO | Two-way Anova  Post hoc: Tukey’s test | - | Figure 4 |
|  |  |  | iNO: 51 | 8 | 8 | 0.523 | - |  |  |  |  |  |
|  |  | PET_CO2_ | CON: 34 | 1 | 7 | - | - | mmHg | CON vs iNO | Two-way Anova  Post hoc: Tukey’s test | - | Figure 4 |
|  |  |  | iNO: 34 | 1 | 7 | 0.277 | - |  |  |  |  |  |
| Question 3. Effect of lowering PASP on basal MSNA | Reducing PASP via iNO, reduced MSNA total activity and burst frequency without reducing burst amplitude | MSNA total activity | CON: 1283 | 484 | 9 | - | - | au·min^-1^ | CON vs iNO | Student T test: Paired (one-tailed) | - | Figure 2 |
|  |  |  | iNO: 996 | 453 | 9 | - | 0.0420 |  |  |  |  |  |
|  |  | MSNA burst frequency | CON: 25 | 8 | 9 | - | - | bursts∙min^-1^ | CON vs iNO | Student T test: Paired (one-tailed) | - | Figure 2 |
|  |  |  | iNO: 21 | 7 | 9 | - | 0.0415 |  |  |  |  |  |
|  |  | MSNA burst amplitude | CON: 51 | 8 | 9 | - | - | au | CON vs iNO | Student T test: Paired (one-tailed) | - | Figure 2 |
|  |  |  | iNO: 46 | 9 | 9 | - | 0.0780 |  |  |  |  |  |
| Question 4.  Effect of lowering PASP on vascular sympathetic baroreflex set-point | Reducing pulmonary artery systolic pressure (PASP) via inhalation of NO (iNO) did not change the MSNA operating-point of the vascular-sympathetic baroreflex. | MSNA burst incidence | CON: 30 | 9 | 9 | - | - | bursts·100HB^-1^ | CON vs iNO | Student T test: Paired (one-tailed) | Table 1 | Figure 5 |
|  |  |  | iNO: 26 | 8 | 9 | - | 0.0730 |  |  |  |  |  |
|  |  | Total MSNA | CON: 1538 | 546 | 9 | - | - | au·100HB^-1^ | CON vs iNO | Student T test: Paired (one-tailed) | - | Figure 5 |
|  |  |  | iNO: 1253 | 584 | 9 | - | 0.0730 |  |  |  |  |  |
|  |  | DBP | CON: 87 | 7 | 9 | - | - | mmHg | CON vs iNO | Two-way Anova  Post hoc: Tukey’s test | - | Figure 5 |
|  |  |  | iNO: 89 | 6 | 9 | 0.507 | - |  |  |  |  |  |
| Question 5.  Effect of reducing PASP on vascular sympathetic reflex responsiveness to transient changes in blood pressure (i.e. gain) | Reducing PASP did not alter spontaneous vascular-sympathetic baroreflex gain | Slope of the linear portion of the relationship between MSNA burst probability and DBP during spontaneous changes in arterial pressure | CON: -2.0 | 0.8 | 8 | - | - | %·mmHg^-1^ | CON vs iNO | Student T test: Paired (two-tailed) | - | Figure 5 |
|  |  |  |  |  |  |  |  |  |  |  |  |  |
|  |  |  | iNO: -3.7 | 1.9 | 8 | - | 0.131 |  |  |  |  |  |
|  |  |  | CON: -90 | 90 | 5 | - | - | au·mmHg^-1^ | CON vs iNO | Student T test: Paired (two-tailed) | - | Figure 5 |
|  |  |  | INO: -173 | 148 | 5 | - | 0.302 |  |  |  |  |  |
| Question 6. Effect of Dopamine on PASP and cardiovascular haemodynamics | Dopamine infusion did not change PASP, peripheral oxygen saturation, heart rate, cardiac output, mean arterial pressure or systemic vascular conductance. | Pulmonary artery systolic pressure (PASP) | CON: 30 | 7 | 8 | - | - | mmHg | CON vs DOPA | Two-way Anova  Post hoc: Tukey’s test | - | Figure 2 |
|  |  |  | DOPA: 30 | 8 | 8 | 0.814 | - |  |  |  |  |  |
|  |  | Peripheral oxygen saturation (SpO_2_) | CON: 84 | 6 | 9 | - | - | % | CON vs DOPA | Two-way Anova  Post hoc: Tukey’s test | - | Figure 4 |
|  |  |  | DOPA: 83 | 6 | 9 | 0.0608 | - |  |  |  |  |  |
|  |  | Heart rate (HR) | CON: 84 | 8 | 9 | - | - | Beats per minute (bpm) | CON vs DOPA | Two-way Anova  Post hoc: Tukey’s test | - | Figure 3 |
|  |  |  | DOPA: 86 | 11 | 9 | 0.120 | - |  |  |  |  |  |
|  |  | Cardiac output (Qc) | CON: 6.1 | 1.6 | 9 | - | - | Litres per min (L·min^-1^) | CON vs DOPA | Two-way Anova  Post hoc: Tukey’s test | - | Figure 3 |
|  |  |  | DOPA: 6.5 | 2.4 | 9 | 0.181 | - |  |  |  |  |  |
|  |  | Mean arterial pressure (MAP) | CON: 105 | 10 | 9 | - | - | mmHg | CON vs DOPA | Two-way Anova  Post hoc: Tukey’s test |  | Figure 3 |
|  |  |  | DOPA: 100 | 9 | 9 | 0.0220 | 0.118 |  |  |  |  |  |
|  |  | Systemic vascular conductance | CON: 5.9 | 1.8 | 9 | - | - | mL·min^-1^·mmHg | CON vs DOPA | Two-way Anova  Post hoc: Tukey’s test | - | Figure 3 |
|  |  |  | DOPA: 6.6 | 2.8 | 9 | 0.0423 | 0.0953 |  |  |  |  |  |
| Question 7. Effect of Dopamine on ventilation | Dopamine infusion reduced ventilation via breathing frequency without influencing end tidal volumes | Ventilation (V_E_) | CON: 26 | 13 | 9 | - | - | Litres per min (L·min^-1^) | CON vs DOPA | Two-way Anova  Post hoc: Tukey’s test | - | Figure 4 |
|  |  |  | DOPA: 14 | 3 | 9 | 0.0343 | P<0.001 |  |  |  |  |  |
|  |  | Tidal volume (V_T_) | CON: 1.7 | 0.5 | 9 | - | - | Litres | CON vs DOPA | Two-way Anova  Post hoc: Tukey’s test | - | Figure 4 |
|  |  |  | DOPA: 1.2 | 0.4 | 9 | 0.0581 | - |  | CON vs DOPA |  |  |  |
|  |  | Breathing frequency (F_B_) | CON: 16 | 5 | 9 | - | - | Breaths per minutes | CON vs DOPA | Two-way Anova  Post hoc: Tukey’s test | - | Figure 4 |
|  |  |  | DOPA: 13 | 3 | 9 | 0.0314 | 0.0178 |  |  |  |  |  |
|  |  | PET_O2_ | CON: 51 | 8 | 8 | - | - | mmHg | CON vs DOPA | Two-way Anova  Post hoc: Tukey’s test | - | Figure 4 |
|  |  |  | DOPA: 51 | 8 | 8 | 0.790 | - |  |  |  |  |  |
|  |  | PET_CO2_ | CON: 34 | 1 | 7 | - | - | mmHg | CON vs DOPA | Two-way Anova  Post hoc: Tukey’s test | - | Figure 4 |
|  |  |  | DOPA: 34 | 1 | 7 | 0.314 | - |  |  |  |  |  |
| Question 8. Effect of blunting the carotid chemoreflex on basal MSNA | Blunting the carotid chemoreflex via dopamine infusion, did not change MSNA total activity, burst frequency and burst amplitude | MSNA total activity | CON: 1283 | 484 | 9 | - | - | au·min^-1^ | CON vs DOPA | Student T test: Paired (two-tailed) | - | Figure 2 |
|  |  |  | DOPA: 1380 | 820 | 9 | - | 0.999 |  |  |  |  |  |
|  |  | MSNA burst frequency | CON: 25 | 8 | 9 | - | - | bursts∙min^-1^ | CON vs DOPA | Student T test: Paired (two-tailed) | - | Figure 2 |
|  |  |  | DOPA: 28 | 13 | 9 | - | 0.112 |  |  |  |  |  |
|  |  | MSNA burst amplitude | CON: 51 | 8 | 9 | - | - | au | CON vs DOPA | Student T test: Paired (two-tailed) | - | Figure 2 |
|  |  |  | DOPA: 47 | 11 | 9 | - | 0.107 |  |  |  |  |  |
| Question 9.  Effect of blunting carotid chemoreflex on vascular sympathetic baroreflex set-point | Blunting carotid chemoreflex via dopamine significantly reduced the MSNA operating-point of the vascular-sympathetic baroreflex. | MSNA burst incidence | CON: 30 | 9 | 9 | - | - | bursts·100HB^-1^ | CON vs DOPA | Student T test: Paired (two-tailed) | Table 1 | Figure 5 |
|  |  |  | DOPA: 33 | 12 | 9 | - | 0.148 |  |  |  |  |  |
|  |  | Total MSNA | CON: 1538 | 546 | 9 | - | - | au·100HB^-1^ | CON vs DOPA | Student T test: Paired (two-tailed) | - | Figure 5 |
|  |  |  | DOPA: 1566 | 773 | 9 | - | 0.420 |  |  |  |  |  |
|  |  | DBP | CON: 87 | 7 | 9 | - | - | mmHg | CON vs DOPA | Two-way Anova  Post hoc: Tukey’s test | - | Figure 5 |
|  |  |  | DOPA: 82 | 9 | 9 | 0.00740 | 0.0211 |  |  |  |  |  |
| Question 10.  Effect of blunting carotid chemoreflex on vascular sympathetic reflex responsiveness to transient changes in blood pressure (i.e. gain) | Reducing carotid chemoreflex did not alter spontaneous vascular-sympathetic baroreflex gain | Slope of the linear portion of the relationship between MSNA burst probability and DBP during spontaneous changes in arterial pressure | CON: -2.0 | 0.8 | 8 | - | - | %·mmHg^-1^ | CON vs DOPA | Student T test: Paired (two-tailed) | - | Figure 5 |
|  |  |  | DOPA: -2.4 | 1.7 | 8 | - | 0.542 |  |  |  |  |  |
|  |  | Slope of the linear portion of the relationship between total MSNA and DBP during spontaneous changes in arterial pressure | CON: -90 | 90 | 5 | - | - | au·mmHg^-1^ | CON vs DOPA | Student T test: Paired (two-tailed) | - | Figure 5 |
|  |  |  | DOPA: -118 | 86 | 5 | - | 0.603 |  |  |  |  |  |
| Question 11. Effect of iNO and dopamine on PASP and cardiovascular haemodynamics | Inhalation of 40ppm nitric oxide and infusion of dopamine significantly reduced PASP with no effect on peripheral oxygen saturation, heart rate, cardiac output, mean arterial pressure or systemic vascular conductance. | Pulmonary artery systolic pressure (PASP) | DOPA: 30 | 87 | 8 | - | - | mmHg | DOPA vs DOPA iNO | Two-way Anova  Post hoc: Tukey’s test | - | Figure 2 |
|  |  |  | DOPA iNO: 22 | 6 | 8 | P<0.001 | 0.0429 |  |  |  |  |  |
|  |  | Peripheral oxygen saturation (SpO_2_) | DOPA: 83 | 6 | 9 | - | - | % | DOPA vs DOPA iNO | Two-way Anova  Post hoc: Tukey’s test | - | Figure 4 |
|  |  |  | DOPA iNO: 21 | 6 | 9 | 0.445 | - |  |  |  |  |  |
|  |  | Heart rate (HR) | DOPA: 86 | 11 | 9 | - | - | Beats per minute (bpm) | DOPA vs DOPA iNO | Two-way Anova  Post hoc: Tukey’s test | - | Figure 3 |
|  |  |  | DOPA iNO: 84 | 9 | 9 | 0.0123 | 0.167 |  |  |  |  |  |
|  |  | Cardiac output (Qc) | DOPA: 6.5 | 2.4 | 9 | - | - | Litres per min (L·min^-1^) | DOPA vs DOPA iNO | Two-way Anova  Post hoc: Tukey’s test | - | Figure 3 |
|  |  |  | DOPA iNO: 6.8 | 2.0 | 9 | 0.671 | - |  |  |  |  |  |
|  |  | Mean arterial pressure (MAP) | DOPA: 100 | 9 | 9 | - | - | mmHg | DOPA vs DOPA iNO | Two-way Anova  Post hoc: Tukey’s test |  | Figure 3 |
|  |  |  | DOPA iNO: 102 | 8 | 9 | 0.0829 | - |  |  |  |  |  |
|  |  | Systemic vascular conductance | DOPA: 6.6 | 2.8 | 9 | - | - | mL·min^-1^·mmHg | DOPA vs DOPA iNO | Two-way Anova  Post hoc: Tukey’s test | - | Figure 3 |
|  |  |  | DOPA iNO: 6.7 | 2.2 | 9 | 0.952 | - |  |  |  |  |  |
| Question 12. Effect of iNO and dopamine on ventilation | Inhalation of 40ppm nitric oxide and dopamine infusion did not change ventilation or end tidal forcing | Ventilation (V_E_) | DOPA: 14 | 3 | 9 | - | - | Litres per min (L·min^-1^) | DOPA vs DOPA iNO | Two-way Anova  Post hoc: Tukey’s test | - | Figure 4 |
|  |  |  | DOPA iNO: 19 | 5 | 9 | 0.190 | - |  |  |  |  |  |
|  |  | Tidal volume (V_T_) | DOPA: 1.2 | 0.3 | 9 | - | - | Litres | DOPA vs DOPA iNO | Two-way Anova  Post hoc: Tukey’s test | - | Figure 4 |
|  |  |  | DOPA iNO: 1.5 | 0.3 | 9 | 0.0843 | - |  |  |  |  |  |
|  |  | Breathing frequency (F_B_) | DOPA: 13 | 3 | 9 | - | - | Breaths per minutes | DOPA vs DOPA iNO | Two-way Anova  Post hoc: Tukey’s test | - | Figure 4 |
|  |  |  | DOPA iNO: 13 | 4 | 9 | 0.667 | - |  |  |  |  |  |
|  |  | PET_O2_ | DOPA: 51 | 8 | 8 | - | - | mmHg | DOPA vs DOPA iNO | Two-way Anova  Post hoc: Tukey’s test | - | Figure 4 |
|  |  |  | DOPA iNO: 51 | 8 | 8 | 0.523 | - |  |  |  |  |  |
|  |  | PET_CO2_ | DOPA: 34 | 1 | 7 | - | - | mmHg | DOPA vs DOPA iNO | Two-way Anova  Post hoc: Tukey’s test | - | Figure 4 |
|  |  |  | DOPA iNO: 34 | 1 | 7 | 0.277 | - |  |  |  |  |  |
| Question 13. Effect of lowering PASP and blunting carotid chemoreflex on basal MSNA | Reducing PASP via iNO and carotid chemoreflex via dopamine, did not change MSNA total activity, burst frequency and burst amplitude | MSNA total activity | DOPA: 1380 | 821 | 9 | - | - | au·min^-1^ | DOPA vs DOPA iNO | Student T test: Paired (one-tailed) | - | Figure 2 |
|  |  |  | DOPA iNO: 1316 | 698 | 9 | - | 0.359 |  |  |  |  |  |
|  |  | MSNA burst frequency | DOPA: 28 | 13 | 9 | - | - | bursts∙min^-1^ | DOPA vs DOPA iNO | Student T test: Paired (one-tailed) | - | Figure 2 |
|  |  |  | DOPA iNO: 26 | 10 | 9 | - | 0.0643 |  |  |  |  |  |
|  |  | MSNA burst amplitude | DOPA: 47 | 11 | 9 | - | - | au | DOPA vs DOPA iNO | Student T test: Paired (one-tailed) | - | Figure 2 |
|  |  |  | DOPA iNO: 48 | 8 | 9 | - | 0.230 |  |  |  |  |  |
| Question 14.  Effect of lowering PASP and blunting the carotid chemoreflex on vascular sympathetic baroreflex set-point | Reducing pulmonary artery systolic pressure (PASP) via inhalation of NO (iNO) and carotid chemoreflex via dopamine did not affect the MSNA operating-point of the vascular-sympathetic baroreflex. | MSNA burst incidence | DOPA: 33 | 12 | 9 | - | - | bursts·100HB^-1^ | DOPA vs DOPA iNO | Student T test: Paired (one-tailed) | Table 1 | Figure 5 |
|  |  |  | DOPA iNO: 31 | 10 | 9 | - | 0.104 |  |  |  |  |  |
|  |  | Total MSNA | DOPA: 1566 | 773 | 9 | - | - | au·100HB^-1^ | DOPA vs DOPA iNO | Student T test: Paired (one-tailed) | - | Figure 5 |
|  |  |  | DOPA iNO: 1546 | 697 | 9 | - | 0.377 |  |  |  |  |  |
|  |  | DBP | DOPA: 82 | 9 | 9 | - | - | mmHg | DOPA vs DOPA iNO | Two-way Anova  Post hoc: Tukey’s test | - | Figure 5 |
|  |  |  | DOPA iNO: 82 | 6 | 9 | 0.507 | - |  |  |  |  |  |
| Question 15.  Effect of reducing PASP and blunting carotid chemoreflex on vascular sympathetic reflex responsiveness to transient changes in blood pressure (i.e. gain) | Reducing PASP blunting the carotid chemoreflexdid not alter spontaneous vascular-sympathetic baroreflex gain | Slope of the linear portion of the relationship between MSNA burst probability and DBP during spontaneous changes in arterial pressure | DOPA: -2.4 | 1.7 | 8 | - | - | %·mmHg^-1^ | DOPA vs DOPA iNO | Student T test: Paired (two-tailed) | - | Figure 5 |
|  |  |  | DOPA iNO: -3.1 | 1.6 | 8 | - | 0.241 |  |  |  |  |  |
|  |  | Slope of the linear portion of the relationship between total MSNA and DBP during spontaneous changes in arterial pressure | DOPA: -118 | 86 | 5 | - | - | au·mmHg^-1^ | DOPA vs DOPA iNO | Student T test: Paired (two-tailed) | - | Figure 5 |
|  |  |  | DOPA iNO: -172 | 128 | 5 | - | 0.403 |  |  |  |  |  |

*You may use multiple lines for the same question to indicate multiple comparisons

** Authors may wish to make the text bold where p is considered significant against a stated confidence limit
